# Supplementary material for: Insights into Haemophilus macrolide resistance: A comprehensive systematic review and meta-analysis
Source: PLoS Negl Trop Dis. 2025 Mar 4;19(3):e0012878. doi: 10.1371/journal.pntd.0012878 (PMC11902202; doi:10.1371/journal.pntd.0012878)
Supplement: S1 Table — Characteristics and extracted data of studies included in the meta-analysis. (DOCX) [file pntd.0012878.s001.docx]

| Author | Year | Countries | Continents | AST method | AST guideline | Year group | Quality group | Species | Erythromycin | Clarithromycin | Azithromycin |
| --- | --- | --- | --- | --- | --- | --- | --- | --- | --- | --- | --- |
| C. T. Atkinson, et al. (1) | 2015 | NA | NA | MIC | EUCAST | 2015_2019 | Low Risk | *H. influenzae* | NA | NA | 7 |
| S. Maddi, et al. (2) | 2017 | United States | Americas | Disk Diffusion | BSAC | 2015_2019 | Low Risk | *H. influenzae* | 20 | 10 | 8 |
| H. J. Wang, et al. (3) | 2019 | China | Asia | Disk Diffusion | CLSI | 2020_2023 | Low Risk | *H. influenzae* | NA | NA | 622 |
| R. S. W. Tsang, et al. (4) | 2017 | Canada | Americas | Disk Diffusion | CLSI | 2015_2019 | Low Risk | *H. influenzae* | NA | 2 | NA |
| C. T. Atkinson, et al. (5) | 2016 | NA | NA | MIC | EUCAST | 2015_2019 | Low Risk | *H. influenzae* | NA | 0 | 4 |
| M. Zhou, et al. (6) | 2021 | China | Asia | Disk Diffusion | CLSI | 2020_2023 | Low Risk | *H. influenzae* | NA | NA | 3341 |
| L. R. Mctaggart, et al. (7) | 2021 | Canada | Americas | MIC | CLSI | 2020_2023 | Low Risk | *H. influenzae* | NA | 26 | NA |
| L. R. Mctaggart, et al. (7) | 2021 | Canada | Americas | MIC | CLSI | 2020_2023 | Low Risk | *H. influenzae* | NA | 8 | NA |
| L. R. Mctaggart, et al. (7) | 2021 | Canada | Americas | MIC | CLSI | 2020_2023 | Low Risk | *H. influenzae* | NA | 91 | NA |
| M. Giufre, et al. (8) | 2018 | Italy | Europe | MIC | EUCAST | 2015_2019 | Low Risk | *H. influenzae* | NA | NA | 15 |
| D. Omoding, et al. (9) | 2019 | NA | NA | Disk Diffusion | CLSI | 2020_2023 | Low Risk | *H. influenzae* | NA | 2 | NA |
| I. L Pez-Hern Ndez, et al. (10) | 2017 | Spain | Europe | MIC | CLSI | 2015_2019 | Low Risk | *H. parainfluenzae* | NA | NA | 2 |
| P. Y. Su, et al. (11) | 2023 | Taiwan | Asia | MIC | CLSI | 2020_2023 | Low Risk | *H. influenzae* | 15 | NA | NA |
| Boroum, et al. (12) | 2015 | Iran | Asia | Disk Diffusion | CLSI | 2015_2019 | Low Risk | *H. influenzae* | NA | NA | 2 |
| L. Roden, et al. (13) | 2019 | NA | NA | NA | NA | 2020_2023 | Low Risk | *H. influenzae* | 32 | NA | NA |
| L. Roden, et al. (13) | 2019 | NA | NA | NA | NA | 2020_2023 | Low Risk | *H. influenzae* | 32 | NA | NA |
| S. Yamada, et al. (14) | 2020 | Japan | Asia | Multiple Method | CLSI | 2020_2023 | Low Risk | *H. influenzae* | NA | 80 | NA |
| M. A. Pfaller, et al. (15) | 2020 | NA | NA | Multiple Method | Multiple Guideline | 2020_2023 | Low Risk | *H. influenzae* | NA | NA | 16 |
| M. A. Pfaller, et al. (15) | 2020 | NA | NA | Multiple Method | Multiple Guideline | 2020_2023 | Low Risk | *H. parainfluenzae* | NA | NA | 1 |

**S1 Table:** Characteristics and extracted data of studies included in the meta-analysis

**NA:** Not applicable.

**References**

1. Atkinson CT, Kunde DA, Tristram SG. Acquired macrolide resistance genes in Haemophilus influenzae? Journal of Antimicrobial Chemotherapy. 2015;70(8):2234-6.

2. Maddi S, Kolsum U, Jackson S, Barraclough R, Maschera B, Simpson KD, et al. Ampicillin resistance in Haemophilus influenzae from COPD patients in the UK. International journal of chronic obstructive pulmonary disease. 2017:1507-18.

3. Wang H-J, Wang C-Q, Hua C-Z, Yu H, Zhang T, Zhang H, et al. Antibiotic resistance profiles of Haemophilus influenzae isolates from children in 2016: a multicenter study in China. Canadian Journal of Infectious Diseases and Medical Microbiology. 2019;2019(1):6456321.

4. Tsang RS, Shuel M, Whyte K, Hoang L, Tyrrell G, Horsman G, et al. Antibiotic susceptibility and molecular analysis of invasive Haemophilus influenzae in Canada, 2007 to 2014. Journal of Antimicrobial Chemotherapy. 2017;72(5):1314-9.

5. Atkinson C, Tristram S. Antimicrobial resistance in cystic fibrosis isolates of Haemophilus influenzae. British journal of biomedical science. 2016;73(2):87-9.

6. Zhou M, Fu P, Fang C, Shang S, Hua C, Jing C, et al. Antimicrobial resistance of Haemophilus influenzae isolates from pediatric hospitals in Mainland China: report from the ISPED program, 2017–2019. Indian Journal of Medical Microbiology. 2021;39(4):434-8.

7. McTaggart LR, Cronin K, Seo CY, Wilson S, Patel SN, Kus JV. Increased incidence of invasive Haemophilus influenzae disease driven by non-type B isolates in Ontario, Canada, 2014 to 2018. Microbiology spectrum. 2021;9(2):e00803-21.

8. Giufrè M, Fabiani M, Cardines R, Riccardo F, Caporali MG, D'Ancona F, et al. Increasing trend in invasive non-typeable Haemophilus influenzae disease and molecular characterization of the isolates, Italy, 2012–2016. Vaccine. 2018;36(45):6615-22.

9. Omoding D, Bazira J. Isolation and antibiotic susceptibility testing of Haemophilus influenzae from nasopharynx of children under five years attending maternal and child health clinic in Mbarara Regional Referral Hospital. canadian journal of infectious diseases and medical microbiology. 2019;2019(1):6542919.

10. López-Hernández I, Machuca J, Diaz de Alba P, Sarvisé C, Gomez F, Rodríguez-Martínez JM, Pascual A. Molecular characterization of fluoroquinolone-resistant Haemophilus parainfluenzae clinical isolates in Spain. Microbial Drug Resistance. 2017;23(8):935-9.

11. Su P-Y, Cheng W-H, Ho C-H. Molecular characterization of multidrug-resistant non-typeable Haemophilus influenzae with high-level resistance to cefuroxime, levofloxacin, and trimethoprim-sulfamethoxazole. BMC microbiology. 2023;23(1):178.

12. Cheng Y, Borum RM, Clark AE, Jin Z, Moore C, Fajtová P, et al. A Dual‐Color Fluorescent Probe Allows Simultaneous Imaging of Main and Papain‐like Proteases of SARS‐CoV‐2‐Infected Cells for Accurate Detection and Rapid Inhibitor Screening. Angewandte Chemie. 2022;134(9):e202113617.

13. Roden L, Görlich D, Omran H, Peters G, Große-Onnebrink J, Kahl BC. A retrospective analysis of the pathogens in the airways of patients with primary ciliary dyskinesia. Respiratory medicine. 2019;156:69-77.

14. Yamada S, Seyama S, Wajima T, Yuzawa Y, Saito M, Tanaka E, Noguchi N. β-Lactamase-non-producing ampicillin-resistant Haemophilus influenzae is acquiring multidrug resistance. Journal of infection and public health. 2020;13(4):497-501.

15. Pfaller MA, Huband MD, Shortridge D, Flamm RK. Surveillance of omadacycline activity tested against clinical isolates from the United States and Europe: report from the SENTRY Antimicrobial Surveillance Program, 2016 to 2018. Antimicrobial agents and chemotherapy. 2020;64(5):10.1128/aac. 02488-19.
