# Supplementary material for: Insights into Haemophilus macrolide resistance: A comprehensive systematic review and meta-analysis
Source: PLoS Negl Trop Dis. 2025 Mar 4;19(3):e0012878. doi: 10.1371/journal.pntd.0012878 (PMC11902202; doi:10.1371/journal.pntd.0012878)
Supplement: S3 Table — Prevalence of Antibiotic Resistance. (DOCX) [file pntd.0012878.s003.docx]

| Antibiotic | Category | Subgroup | K (n, N) | Proportion 95%CI(LCI, HCI) | I² | P1 | P2 | P3 |
| --- | --- | --- | --- | --- | --- | --- | --- | --- |
| Erythromycin | Overall | NA | 4 (99, 133) | 0.790 (0.509, 0.932) | 85.84% | p=0.044 | p<0.001 | NA |
|  | Continents | Americas | 1 (20, 39) | 0.513 (0.360, 0.664) | 0.00% | p=0.873 | p>0.999 | p<0.001 |
|  |  | Asia | 1 (15, 26) | 0.577 (0.385, 0.748) | 0.00% | p=0.435 | p>0.999 |  |
|  |  | NA | 2 (64, 68) | 0.941 (0.854, 0.978) | 0.00% | p<0.001 | p>0.999 |  |
|  | AST method | Disk Diffusion | 1 (20, 39) | 0.513 (0.360, 0.664) | 0.00% | p=0.873 | p>0.999 | p<0.001 |
|  |  | MIC | 1 (15, 26) | 0.577 (0.385, 0.748) | 0.00% | p=0.435 | p>0.999 |  |
|  |  | NA | 2 (64, 68) | 0.941 (0.854, 0.978) | 0.00% | p<0.001 | p>0.999 |  |
|  | AST guideline | BSAC | 1 (20, 39) | 0.513 (0.360, 0.664) | 0.00% | p=0.873 | p>0.999 | p<0.001 |
|  |  | CLSI | 1 (15, 26) | 0.577 (0.385, 0.748) | 0.00% | p=0.435 | p>0.999 |  |
|  |  | NA | 2 (64, 68) | 0.941 (0.854, 0.978) | 0.00% | p<0.001 | p>0.999 |  |
|  | Year group | 2015_2019 | 1 (20, 39) | 0.513 (0.360, 0.664) | 0.00% | p=0.873 | p>0.999 | p=0.314 |
|  |  | 2020_2023 | 3 (79, 94) | 0.867 (0.506, 0.976) | 86.04% | p=0.047 | p<0.001 |  |
| Clarithromycin | Overall | NA | 7 (219, 2700) | 0.072 (0.029, 0.169) | 95.86% | p<0.001 | p<0.001 | NA |
|  | Countries | United States | 1 (10, 39) | 0.256 (0.144, 0.414) | 0.00% | p=0.004 | p>0.999 | p=0.502 |
|  |  | Canada | 4 (127, 1998) | 0.070 (0.014, 0.275) | 96.91% | p=0.002 | p<0.001 |  |
|  |  | Japan | 1 (80, 260) | 0.308 (0.255, 0.366) | 0.00% | p<0.001 | p>0.999 |  |
|  | Continents | Americas | 5 (137, 2037) | 0.094 (0.027, 0.279) | 96.15% | p<0.001 | p<0.001 | p=0.049 |
|  |  | NA | 2 (2, 403) | 0.007 (0.002, 0.023) | 0.00% | p<0.001 | p=0.558 |  |
|  |  | Asia | 1 (80, 260) | 0.308 (0.255, 0.366) | 0.00% | p<0.001 | p>0.999 |  |
|  | AST method | Disk Diffusion | 3 (14, 1169) | 0.019 (0.001, 0.357) | 96.12% | p=0.021 | p<0.001 | p=0.266 |
|  |  | MIC | 4 (125, 1271) | 0.100 (0.024, 0.333) | 95.78% | p=0.004 | p<0.001 |  |
|  |  | Multiple Method | 1 (80, 260) | 0.308 (0.255, 0.366) | 0.00% | p<0.001 | p>0.999 |  |
|  | AST guideline | BSAC | 1 (10, 39) | 0.256 (0.144, 0.414) | 0.00% | p=0.004 | p>0.999 | p=0.140 |
|  |  | CLSI | 6 (209, 2506) | 0.072 (0.025, 0.190) | 97.12% | p<0.001 | p<0.001 |  |
|  |  | EUCAST | 1 (0, 155) | 0.003 (0.000, 0.049) | 0.00% | p<0.001 | p>0.999 |  |
|  | Year group | 2015_2019 | 3 (12, 1076) | 0.015 (0.000, 0.428) | 95.64% | p=0.035 | p<0.001 | p=0.080 |
|  |  | 2020_2023 | 5 (207, 1624) | 0.126 (0.047, 0.296) | 97.02% | p<0.001 | p<0.001 |  |
| Azithromycin | Overall | NA | 9 (4018, 13069) | 0.093 (0.056, 0.150) | 96.31% | p<0.001 | p<0.001 | NA |
|  | Countries | United States | 1 (8, 39) | 0.205 (0.106, 0.360) | 0.00% | p<0.001 | p>0.999 | p<0.001 |
|  |  | China | 2 (3963, 10817) | 0.340 (0.265, 0.425) | 97.93% | p<0.001 | p<0.001 |  |
|  |  | Italy | 1 (15, 263) | 0.057 (0.035, 0.092) | 0.00% | p<0.001 | p>0.999 |  |
|  |  | Spain | 1 (2, 12) | 0.167 (0.042, 0.477) | 0.00% | p=0.038 | p>0.999 |  |
|  |  | Iran | 1 (2, 20) | 0.100 (0.025, 0.324) | 0.00% | p=0.003 | p>0.999 |  |
|  | Continents | NA | 4 (28, 1918) | 0.024 (0.010, 0.058) | 75.24% | p<0.001 | p=0.007 | p<0.001 |
|  |  | Americas | 1 (8, 39) | 0.205 (0.106, 0.360) | 0.00% | p<0.001 | p>0.999 |  |
|  |  | Asia | 3 (3965, 10837) | 0.322 (0.248, 0.405) | 96.24% | p<0.001 | p<0.001 |  |
|  |  | Europe | 2 (17, 275) | 0.081 (0.029, 0.208) | 53.11% | p<0.001 | p=0.144 |  |
|  | AST method | MIC | 4 (28, 602) | 0.050 (0.029, 0.085) | 44.37% | p<0.001 | p=0.145 | p<0.001 |
|  |  | Disk Diffusion | 4 (3973, 10876) | 0.304 (0.236, 0.381) | 94.77% | p<0.001 | p<0.001 |  |
|  |  | Multiple Method | 2 (17, 1591) | 0.020 (0.003, 0.104) | 67.73% | p<0.001 | p=0.078 |  |
|  | AST guideline | EUCAST | 3 (26, 590) | 0.045 (0.030, 0.068) | 10.50% | p<0.001 | p=0.327 | p<0.001 |
|  |  | BSAC | 1 (8, 39) | 0.205 (0.106, 0.360) | 0.00% | p<0.001 | p>0.999 |  |
|  |  | CLSI | 4 (3967, 10849) | 0.312 (0.242, 0.393) | 94.55% | p<0.001 | p<0.001 |  |
|  |  | Multiple Guideline | 2 (17, 1591) | 0.020 (0.003, 0.104) | 67.73% | p<0.001 | p=0.078 |  |
|  | Year group | 2015_2019 | 6 (38, 661) | 0.074 (0.038, 0.140) | 73.16% | p<0.001 | p=0.002 | p=0.218 |
|  |  | 2020_2023 | 4 (3980, 12408) | 0.127 (0.067, 0.228) | 99.03% | p<0.001 | p<0.001 |  |
|  | Species | *H. influenzae* | 8 (4015, 13041) | 0.091 (0.053, 0.151) | 98.49% | p<0.001 | p<0.001 | p=0.785 |
|  |  | *H. parainfluenzae* | 2 (3, 28) | 0.119 (0.038, 0.312) | 0.00% | p=0.001 | p=0.395 |  |
| Caption; K: Number of reports, n: Number of resistant isolates, N: Number of total isolates, LCI: 95% Lower Limit Confidence Interval, HCI: 95% Higher Limit Confidence Interval, P1: P-value of difference from zero resistance rate, P2: P-value of heterogeneity between reports, P3: P-value of difference between groups. | | | | | | | | |

S3 Table: Prevalence of Antibiotic Resistance
