# Supplementary material for: Insights into Haemophilus macrolide resistance: A comprehensive systematic review and meta-analysis
Source: PLoS Negl Trop Dis. 2025 Mar 4;19(3):e0012878. doi: 10.1371/journal.pntd.0012878 (PMC11902202; doi:10.1371/journal.pntd.0012878)
Supplement: Attachment — Submitted filename: comments.docx [file pntd.0012878.s004.docx]

I appreciate the invitation to review this study; thank you for including me. I’ve expressed my gratitude to the authors. The study is well-structured, but some suggestions could enhance its quality:

- Remove the heading for the Objective from the abstract.

- Eliminate the repetition in lines 33-34.

- Replace “nine” with the digit “9” and “four” with the digit “4” in line 46.

- Combine Section 2.3 into a single paragraph; remove the paragraph mark in line 130.

- Move lines 176-178 to the next section.

- Provide more detailed discussion of AST results related to the major species (*H. influenzae* and *H. parainfluenzae*) investigated in this study.

- In line 365, add a sentence summarizing future research directions, such as “Further studies should investigate the impact of resistance on clinical outcomes and alternative therapeutic strategies.”
