# Supplementary material for: Insights into Haemophilus macrolide resistance: A comprehensive systematic review and meta-analysis
Source: PLoS Negl Trop Dis. 2025 Mar 4;19(3):e0012878. doi: 10.1371/journal.pntd.0012878 (PMC11902202; doi:10.1371/journal.pntd.0012878)
Supplement: Attachment — Submitted filename: Response to Reviewers.docx [file pntd.0012878.s006.docx]

**Dear Reviewer**

First, we would like to thank you for your meticulous review of our paper and for your valuable comments, corrections, and suggestions. All the revised sections in the manuscript have been highlighted in yellow for your convenience. We hope these changes enhance our study's clarity and comprehensibility and address your concerns effectively.

Once again, we sincerely thank you for your diligent review, and we are committed to ensuring that our manuscript meets the highest standards of quality and accuracy.

Here are my corrections/suggestions:

Reviewer #4: 1. Abstract, Line 51: “Clarithromycin resistance increased from q.5% (2015–2019) to 12.6% (2020–2023)”. Please check the number q.5%

**Response:** **The value has been reviewed and corrected to the accurate percentage in Line 47 of the revised abstract.**

2. Selection Process, Lines 129-130: “Following a systematic database search, duplicates were removed using EndNote (version 21), and duplicate entries were removed”. Rewrite sentence to remove redundancy.

**Response:** **The text has been revised to eliminate redundancy and improve clarity. The updated sentence can be found on Line 173-174.**

3. Section 2.4 and 2.5: Information in both these sections seems redundant? Can this be merged?
**Response:** **We have addressed this concern by merging the content from Sections 2.4 and 2.5 into a single section, now titled Section 2.4. This revision eliminates redundancy and ensures clarity. The updated section can be found on lines 178-181.**

4. Line 151-152: “S scores between 4 and 6 signified some concern, whereas scores less than 4 indicated low quality”. The first S seems like a typo. Please correct if that’s the case.
**Response:** **The text has been reviewed, and the typo has been corrected on line 193.**

5. Section 3.1 Lines 176-127: “These studies originated from eight countries (the United States, China, Canada, Italy, Spain, Taiwan, Iran, and Japan) spanning four continents (North America, America, Asia, and Europe). The included studies will be conducted between 2015 and 2023” The countries listed span only three continents. America is not a continent. Please correct since North America is already included.

**Response: corrected, lines 234-235.**

6. Section 3.2 Lines 184-187: “Among the guidelines used to interpret antimicrobial susceptibilities, CLSI (Clinical & Laboratory Standards Institute), EUCAST (European Committee on Antimicrobial Susceptibility Testing), and BSAC (The British Society for Antimicrobial Chemotherapy) were widely used in most studies (12, four, and one reports, respectively)”. Please be consistent in the usage of how the numbers are depicted i.e., 12 vs. four and one).

**Response: All the numbers have been written in words for clarity and uniformity. This correction has been made on Line 244.**

Figure 2: There is footnote embedded in the figure. Please include that in the legend of the Figure.

**Response:** **We have incorporated the content of the footnote into the figure legend for improved clarity and consistency. The revised legend for Figure 2 now reads as follows:**

**"Each antibiotic's resistance proportion is represented by a box plot, with error bars indicating 95% confidence intervals. Individual study results are depicted as red points within each category, while the diamond shape at the bottom of each section represents the overall pooled estimate. Heterogeneity statistics (I² and p-values) are also included, offering insights into variability across the included studies. This visualization highlights the variability in resistance rates across studies and the aggregated estimate for each antibiotic."**

8. Section 3.4.1 Lines 237-243 and Figure 5A: Since the data for North America is being discussed it would be best if the America’s data (which I assume is North and South America combined) be split to be able to relate the text to Figure 5A.

**Response:** **We would like to clarify that the dataset used in our study does not include data from South America; all data labeled under "Americas" pertains exclusively to North America. To avoid any potential misunderstanding, we have revised the text in Section 3.4.1 (line 295) and updated Figure 5A to state "Americas (North America) explicitly.**

9. Figure 5 was extremely hard to review because of the quality of the figure. Please ensure that the high-resolution figure gets published in the final manuscript. Usually, there is an option to download high-resolution figures during the review process, which I believe was not provided here.

**Response: The low quality of Figure 5 may have resulted from the conversion to a PDF file during submission. We have ensured that the figure is now in high resolution and will confirm that it is submitted and published in its highest quality in the final manuscript.**

10. Discussion Lines 328-331: “A meta-regression analysis explored the relationship between macrolide resistance rates and publication years and revealed a statistically significant correlation. This finding suggests that macrolide resistance in Haemophilus spp. May increase, aligning with the global trend of rising antimicrobial resistance (43).” I think it will be worth highlighting that a correlation was only observed for one (Clarithromycin) of the three antibiotics tested.

**Response:** **We have revised the text in Lines 427-431 to clarify that the observed correlation was specific to clarithromycin and that no significant correlations were identified for the other two macrolides tested.**

11. Please discuss the limitations of the type of meta-analysis performed in this study. For example, what if there is resistance to a particular macrolide in a region but that has never been studied. How do we account for that? Please add additional limitations as deemed fit.

**Response: In response to your suggestion, we have expanded the discussion of our study’s limitations in Lines 467–476. Specifically, we acknowledge that our meta-analysis depends on published data, which inherently limits the representation of resistance rates in regions where relevant studies have not been conducted. This creates a potential gap in understanding resistance patterns in underrepresented or unstudied areas. Additionally, we discuss the possible influence of geographic and reporting biases, as well as the variability introduced by differences in study design, sample sizes, and methodologies across the included studies. Moreover, we address the limitations associated with the temporal nature of the data, as certain included studies may not accurately reflect current resistance trends due to time lags.**

**Dear Reviewer**

First, we would like to thank you for your very careful review of our paper and for your subsequent comments, corrections, and suggestions as well. All the revised parts in the manuscript file are highlighted in red.

Reviewer #2: The study’s focus on clarithromycin, azithromycin, and erythromycin excludes newer or less commonly used macrolides, potentially limiting the comprehensive understanding of resistance dynamics. A more inclusive approach would involve investigating macrolides and, if applicable, other antibiotics from the same class. This should not be restricted to specific antibiotics.

**Response: Thank you for your insightful comment. We want to clarify that our study did not intentionally exclude any macrolide antibiotics. Our search strategy was designed to be comprehensive and inclusive, as demonstrated by the search syntax provided:**

**("Erythromycin" OR "Clarithromycin" OR "Azithromycin" OR "Dirithromycin" OR "Spiramycin" OR "Josamycin" OR "Roxithromycin" OR "Troleandomycin" OR "Clindamycin" OR "Telithromycin" OR "macrolide").**

**This search included all macrolides and relevant antibiotics available for Haemophilus spp. resistance studies. However, based on the search results, the data available from the literature were restricted to erythromycin, clarithromycin, and azithromycin. No resistance data were found for the other macrolides or related antibiotics for this bacterial species. Therefore, our analysis reflects the scope of the available data rather than a study design limitation.**

Differences in resistance rates between AST methods and guidelines are noted but not adequately contextualized in terms of their implications for clinical practice and policymaking.
Provide detailed recommendations for standardizing AST methods to reduce variability and enhance comparability of resistance data.

**Response:**  **We have addressed your concern by elaborating on the importance of standardizing AST methods and harmonizing guidelines in the manuscript. In the revised text (Lines 403-426), we discuss how** **variability in AST methods, such as Disk Diffusion, molecular methods, and broth microdilution, as well as differences in guidelines like CLSI and EUCAST, can lead to inconsistencies in resistance detection and data interpretation. These discrepancies have significant implications for clinical practice and policymaking, potentially leading to inappropriate antibiotic selection and hindering effective public health interventions. To mitigate these challenges, we provide detailed recommendations, including adopting internationally recognized guidelines, harmonizing breakpoints, strict** **quality control measures, integrating molecular testing, and global collaboration through centralized databases and training initiatives.**

**Additionally, we highlight our study's findings from the meta-regression analysis, which revealed a statistically significant correlation for clarithromycin resistance rates over time, while no significant correlation was observed for the other two macrolides. This aligns with the global trend of rising antimicrobial resistance and emphasizes the critical need for standardized methods to ensure reliable resistance surveillance. We appreciate your suggestion, which has enhanced the clarity and depth of the manuscript.**

Although mechanisms such as erm and mefE genes are briefly mentioned, the discussion could benefit from a deeper exploration of genetic and molecular factors driving macrolide resistance.

**Response: We expanded the discussion in Lines 93–111 to provide a more detailed explanation of resistance mechanisms in Haemophilus spp., including ribosomal target modification via erm genes, efflux pump activity driven by mefE, and the role of mobile genetic elements in horizontal gene transfer. We also highlighted biofilm formation as a barrier to antibiotic penetration and its synergy with efflux pumps like AcrB in promoting multidrug resistance. These mechanisms collectively underscore the challenges in treating infections caused by Haemophilus spp.**

Line 313: Minor grammatical errors and awkward phrasing (e.g., “red resistance rates”) should be corrected for clarity.

**Response: We have corrected the grammatical errors and clarified the phrasing to improve clarity. The changes are implemented in line 372 of the revised manuscript.**

Line 34: Replace “pooled random-effects model in R, utilizing the metafor package” with “random-effects model using the metafor package in R” for conciseness.

**Response: We have revised the phrasing for improved conciseness, updating the text on lines 35-36 to read: “random-effects model using the metafor package in R.**
Line 52: The sentence “The high prevalence and regional variability of resistance highlight…” is slightly repetitive of earlier content. Revise to emphasize the findings’ implications.

**Response: We have revised the sentence on Line 52 to remove redundancy and rephrased it to emphasize the implications of the findings more effectively.**

Line 69-71: Replace “thereby reducing the binding affinity of macrolides” with “leading to decreased macrolide efficacy” for brevity.

**Response: This was corrected**.
Line 114-116: Rephrase “studies with sample sizes of less than three isolates and antibiotics with fewer than three isolates were excluded” for clarity. Consider splitting it into two sentences.

**Response: We have revised the text on lines 159-160 to improve clarity. The updated text now reads: "Studies with sample sizes of fewer than three isolates were excluded from the analysis. Similarly, antibiotics tested in fewer than three isolates were also excluded.**

Line 236-243 (Subgroup Analysis by Region): Provide additional context for resistance variability, including differences in healthcare practices or antibiotic use policies.

**Response: We have revised Lines 290–307 to include a more detailed discussion of the potential factors driving regional differences in resistance rates. Specifically, we now highlight how variations in healthcare infrastructure, antibiotic prescribing patterns, antimicrobial stewardship programs, and the availability of antibiotics over the counter may contribute to the observed discrepancies in resistance prevalence.**

**Furthermore, we emphasize the role of stricter antibiotic usage policies and robust surveillance systems in reducing resistance rates, contrasting this with the challenges faced in regions with high antibiotic consumption and less regulation. These additions aim to provide a more comprehensive understanding of global contextual factors influencing resistance trends. We appreciate your suggestion, which has strengthened the interpretation of our findings and their implications for public health interventions.**

Line 301-304: Include a specific example illustrating how “globalization of travel and trade” may contribute to the spread of resistance.

**Response:** **We have incorporated a specific example in Lines 377–382 to illustrate how the “globalization of travel and trade” contributes to the spread of antimicrobial resistance. This example focuses on the transmission of resistant strains of *Haemophilus* spp, via international travel and the global trade of agricultural products. By including this detailed instance, we aim to clarify the connection between globalization and the dissemination of resistance while highlighting the importance of international collaboration in monitoring and addressing antimicrobial resistance.**

Line 328-329: Rephrase “a statistically significant correlation” as “a significant positive trend” to align with the tone of the remainder of the discussion.

**Response: This change has been implemented on line 339.**
Line 337-340: Expand on the “implications for public health policies” with specific recommendations for policy-makers.

**Response: We have revised Lines 403–426 to include detailed and actionable recommendations. These additions emphasize the importance of establishing standardized guidelines for antimicrobial susceptibility testing, strengthening resistance surveillance systems, and implementing stricter regulations on over-the-counter antibiotic sales. Furthermore, we have highlighted the need for international collaboration to track resistant strains and harmonize antimicrobial use policies globally.**

References: Ensure consistent formatting of journal names and volume/issue numbers.

**Response: We have reviewed the references and ensured that the formatting of journal names, volume numbers, and issue numbers is consistent throughout the manuscript.**

Tables: Supplementary tables should be cross-referenced in the main text for ease of access.

**Response:** **We confirm that all three supplementary tables (S1 Table, S2 Table, and S3 Table) have been explicitly and appropriately cross-referenced within the main text to facilitate ease of access for readers.**

Figure 3: Labels have been added to clarify the axes for readers unfamiliar with meta-analysis visualizations.
Use more intuitive and comprehensive captions to figures.

**Response: We have carefully revised and expanded the captions for all figures, including Figure 3, to ensure they provide clear, intuitive, and comprehensive explanations.**

**Dear Reviewer**

First, we would like to thank you for your meticulous review of our paper and for your subsequent comments, corrections, and suggestions. All the revised parts in the manuscript file are highlighted in green.

Reviewer #3: This study evaluates macrolide resistance in Haemophilus species globally through a systematic review and meta-analysis. It identifies significant resistance trends, highlights regional variability, and emphasizes the need for localized treatment strategies and enhanced surveillance. Advanced statistical methods, including meta-regression and random-effect modeling, are employed to ensure reliable conclusions. The study’s adherence to PRISMA guidelines and PROSPERO registration ensures transparency and reproducibility, making it a valuable resource for addressing antimicrobial resistance challenges, especially in underrepresented regions.
There are several comments and suggestions for improvement outlined below to enhance the study's comprehensiveness and impact.
Comments for Improvement
General Comments
1. Study Scope and Limitations:
Although a comprehensive search strategy was performed, the inclusion of only 15 studies is a notable limitation. This could affect the generalizability of findings, especially for underrepresented regions like Africa and South America where data were sparse or missing. Please acknowledge this limitation explicitly in the manuscript and discuss its implications.
The focus on clarithromycin, azithromycin, and erythromycin excludes newer or less commonly used macrolides. Expanding the analysis to include additional macrolides or related antibiotics would provide a more comprehensive understanding of resistance dynamics.

**Response: Thank you for your insightful comment. We want to clarify that our study did not intentionally exclude macrolide antibiotics. Regarding the focus on clarithromycin, azithromycin, and erythromycin, we have clarified in the manuscript that our analysis included all macrolides reported in the available literature. However, no data were identified for newer or less commonly used macrolides in the context of Haemophilus spp., which reflects the current limitations in the published evidence. Our search strategy was designed to be comprehensive and inclusive, as demonstrated by the search syntax provided:**

**("Erythromycin" OR "Clarithromycin" OR "Azithromycin" OR "Dirithromycin" OR "Spiramycin" OR "Josamycin" OR "Roxithromycin" OR "Troleandomycin" OR "Clindamycin" OR "Telithromycin" OR "macrolide").**

**This search included all macrolides and relevant antibiotics available for *Haemophilus* spp—resistance studies. However, based on the search results, the data available from the literature were restricted to erythromycin, clarithromycin, and azithromycin. No resistance data were found for the other macrolides or related antibiotics for this bacterial species. Therefore, our analysis reflects the scope of the available data rather than a study design limitation.**

**We have explicitly acknowledged this limitation in Lines 477–484 of the manuscript. Specifically, we discuss how the inclusion of only 15 studies may impact the generalizability of our findings, particularly for underrepresented regions such as Africa and South America, where data were sparse or missing. Additionally, we emphasize the need for enhanced surveillance and reporting in these regions to provide a more comprehensive understanding of macrolide resistance and to inform targeted public health interventions.**

2. AST Methods and Clinical Implications: Differences in resistance rates across antimicrobial susceptibility testing (AST) methods and guidelines are noted but not adequately contextualized. Discuss how these differences impact clinical decision-making and policymaking.

**Response: We have addressed your concern by elaborating on the importance of standardizing AST methods and harmonizing guidelines in the manuscript. In the revised text (Lines 403-426), we discuss how variability in AST methods, such as Disk Diffusion, molecular techniques, and broth microdilution, as well as differences in guidelines like CLSI and EUCAST, can lead to inconsistencies in resistance detection and data interpretation. These discrepancies have significant implications for clinical practice and policymaking, potentially leading to inappropriate antibiotic selection and hindering effective public health interventions. To mitigate these challenges, we provide detailed recommendations, including adopting internationally recognized guidelines, harmonizing breakpoints, strict quality control measures, integrating molecular testing, and global collaboration through centralized databases and training initiatives.**

3. Species-Specific Discussion:

The inclusion of Haemophilus influenzae and Haemophilus parainfluenzae is valuable, but these species are not sufficiently discussed in the introduction and discussion. Provide more context about their relevance and resistance trends, particularly since the resistance comparison for azithromycin is limited due to data constraints.

**Response: We have expanded the Introduction (Lines 72-80) and Discussion (Lines 346–352) to provide additional context about the clinical relevance of these species and their resistance trends.**
Methodological Clarifications
1. Clearly define and justify the study’s definition of terms and reporting criteria in the Methods section.

**Response: We have revised Lines 208-216 to explicitly define the terms study and report and explain their application in this analysis. Specifically, a study is defined as a single article, while a report refers to individual datasets or analyses within a study. Each study could include one or more reports, corresponding to distinct subpopulations, antibiotics, periods, or methodologies described in the article. To ensure consistency, data from all reports within a study were aggregated for study-level analyses while preserving the independence of findings across studies.**

2. Provide additional details about statistical techniques used, such as back transformation, double arcsin transformation, and logistic regression, to enhance transparency.

**Response: We have revised the section on statistics (Lines 220-221) to include additional details regarding the methods used. Specifically, we have clarified the application of the double arcsine transformation method, which stabilized variance and normalized** **proportions for meta-analysis.**

3. For the Risk of Bias Assessment (Lines 146-152), clarify how "some concern" and "low quality" ratings were classified, and include thresholds for these classifications.

**Response: Thank you for commenting on the classification of "some concern" and "low quality" ratings in the Risk of Bias Assessment. In response, we have clarified this in Lines 189-195 by explicitly detailing the thresholds for each classification. Specifically, we defined the scoring system using numerical values assigned to each risk level (low risk, some concern, and high risk). We outlined the criteria for categorizing studies as high quality (scores >7), some concern (scores between 4 and 6), and low quality (scores <4).**

Minor Comments

1. Remove unnecessary lines, such as Lines 22-23.

**Response: We have carefully reviewed the manuscript and removed the unnecessary lines.**
2. Lines 33-34 are repeated from the Methods section; please remove them.

**Response: The repeated lines have been identified and removed as suggested.**
3. Add Haemophilus influenzae as a keyword to improve indexing.

**Response: The keyword Haemophilus influenzae has now been added as requested.**
4. Ensure consistent font color throughout the manuscript (use black for all text).

**Response: We have ensured that the font color is consistent throughout the manuscript, with black being used for all text.**
5. Line 187: Adjust numerical formatting to ensure consistency.

**Response: The numerical formatting issue has been addressed and corrected. The updated formatting can be found on line 244 of the revised manuscript.**

6. Line 291: Verify and correct reference formatting.

**Response: We have reviewed and corrected the reference formatting, which is now updated on line 362 as reference 47**

7. Line 324: Add appropriate spacing before and after Reference 41.

**Response: Was corrected**
8. Lines 325-326: Use only abbreviations for the Clinical and Laboratory Standards Institute (CLSI) and the European Committee on Antimicrobial Susceptibility Testing (EUCAST) after first mention.

**Response: This adjustment has been made on line 401.**
